# Monocrotophos-Induced Intermediate Syndrome Complicated by Hyperammonemic Encephalopathy: A Case Report

**DOI:** 10.7759/cureus.48527

**Published:** 2023-11-08

**Authors:** Bijit Bharali, Naveen Kumaran, Durga Shankar Meena, Mahendra Kumar Garg

**Affiliations:** 1 Internal Medicine, All India Institute of Medical Sciences, Jodhpur, Jodhpur, IND

**Keywords:** intermediate syndrome, altered sensorium, hyperammonemic encephalopathy, monocrotophos, organophosphorus poisoning

## Abstract

Organophosphorus (OP) poisoning is the most common type of poisoning in India. Amongst the OP, monocrotophos poisoning has the highest lethality and need for mechanical ventilation. Monocrotophos is also implicated in causing OP-induced intermediate syndrome, the prevalence of which is 10-40% of all OP poisoning. The other neurological manifestations are delayed neuropathy and neuropsychiatric syndrome. We herein discuss a case of a 58-year-old male who presented with monocrotophos poisoning and intermediate syndrome. During the hospitalisation course, the patient developed hyperammonemic encephalopathy, resulting in difficulty in weaning from mechanical ventilation. After ruling out all possible causes of hyperammonemia, it was attributed to monocrotophos poisoning. The patient improved significantly after initiating lactulose and was successfully weaned off from the ventilator. This report highlights the high index of suspicion of hyperammonemic encephalopathy in monocrotophos toxicity, which can be easily missed due to other commoner neurological manifestations of organophosphorus poisoning.

## Introduction

Organophosphorus (OP) poisoning continues to be the most frequent cause of self-poisoning, especially in the developing world [1]. In the Indian subcontinent, more than 30% of suicidal deaths are attributed to organophosphorus poisoning [2]. There is a wide array of neurological syndromes described in OP toxicity, like impaired consciousness due to acute cholinergic crisis, intermediate syndrome with involvement of respiratory muscles, and OP-induced delayed polyneuropathy (OPIDP) [3]. Occasionally, chronic OP exposure may reflect as neuropsychiatric symptoms also. Monocrotophos are highly notorious compounds that cause the intermediate syndrome. Monocrotophos is a methylamine derivative that can lead to hyperammonemia and precipitate metabolic encephalopathy on acute heavy exposure. It can significantly impact the mortality and morbidity of such cases, which is usually a reversible and preventable cause of metabolic encephalopathy. We herein report a case of severe monocrotophos toxicity-induced intermediate syndrome with hyperammonemic encephalopathy leading to ventilator weaning failure. This case discusses the importance of early recognition and prevention of metabolic encephalopathy in patients with monocrotophos poisoning.

## Case presentation

A 58-year-old male, a farmer by occupation, with a known case of type 2 diabetes mellitus, presented to the emergency room with an alleged history of monocrotophos consumption (amount consumed around 20-30 mL). The patient was found unconscious with frothing in his mouth since morning. He was managed with gastric lavage, pralidoxime (10 mL/hour), antiemetics, and laxatives in a nearby hospital; subsequently, he was referred to our centre for further management. Upon arrival to the emergency room, the patient's vitals were: blood pressure 160/90 mmHg, pulse rate 120/min, respiratory rate 16/min, and SPO2 84% at room air. Blood gas analysis showed metabolic acidosis with lactic acidemia. On examination patient's GCS (Glasgow coma scale) was E2V1M4, and bilateral pinpoint pupils and frothing from the mouth were also noted. Chest auscultation revealed bilateral coarse crepitations. To secure the airway patient was intubated using 7.5 mm ETT (endotracheal tube) after induction with etomidate (50mg). The patient's Peradeniya score at presentation was 5 (pinpoint pupil - 2, generalized fasciculations - 1, no verbal response - 2), and therefore, he was diagnosed with moderate OP poisoning [4]. Subsequently, the patient was administered atropine initially at 1.2 mg bolus. The later doses were doubled at an interval of 2 mins; atropinization was achieved at 4.8 mg atropine dose. Subsequently, an atropine infusion was initiated (0.8 mg/hour). Atropine infusion was tapered and stopped on post-poisoning day 3.

On day 4 of hospitalisation, central nervous system (CNS) examination was repeated, revealing bilaterally reduced muscle tone (in all four limbs), decreased power of distal muscles of left upper and lower limbs, and proximal plus distal muscles of the right upper and lower limb. Deep tendon reflexes were absent bilaterally, and the plantar reflex was mute on the left side. Neck holding time was also reduced (4 seconds). Neurology consultation was taken and advised for contrast-enhanced MRI Brain, electroencephalogram (EEG), and nerve conduction study (NCS). MRI brain was reported to have sequelae of old infarct with gliosis and haemorrhage in the left basal ganglia with mild exvacuodilation of the left lateral ventricle and small vessel ischemic changes. EEG showed moderate cerebral dysfunction without epileptiform discharges. NCS of left upper and lower limb suggestive of motor axonal large fibre neuropathy. Therefore the patient was diagnosed with OP-induced intermediate syndrome. The patient later developed two fever spikes for which urine, tracheal secretions, and blood cultures were sent for culture, which were found to be sterile, and piperacillin-tazobactam was added empirically. The patient became afebrile within 48 hours of starting antibiotics.

On post-poisoning day 9, the patient's GCS dropped to E2VTM3; however, repeat non-contrast CT (NCCT) brain revealed no acute changes. Cerebrospinal fluid (CSF) analysis showed six white blood cells and mildly elevated protein (85, 30-45 mg/dl). Biochemical investigations revealed mild hypernatremia (147, 135-145 mEq/L), for which 0.45% saline and free water correction started. Serum sodium was normalised without improvement in his consciousness. The other causes of altered sensorium were also ruled out, like sepsis or uremic encephalopathy. Notwithstanding, the patient also had constipation for the last six days. In view of altered sensorium and persistent constipation, serum ammonia levels were sent, which were found to be highly elevated ( 386.2; 17-90 µg/dl). However, nothing was pointing toward hepatic encephalopathy in history and lab diagnosis. No evidence of chronic liver disease or portal hypertension was found in the ultrasound whole abdomen. Measures for hyperammonemic encephalopathy were initiated with oral lactulose, per rectal lactulose enema, oral rifaximin, and L-ornithine L-aspartate (LOLA). Repeat serum ammonia levels after 48 hours of treatment initiation showed a declining trend (225 µg/dl). The patient's GCS improved to E4VTM6, and subsequently, a weaning trial was given on spontaneous mode ventilation with positive end-expiratory pressure (PEEP) of 6 cm H2O and pressure support of 8 cm H2O. After tolerating spontaneous mode ventilation for 24 hours, the patient was successfully extubated with the normalisation of serum ammonia. 

On post-poisoning day 21, CNS examination was repeated, which showed GCS of E4V3M6 and the pupils were equal (4mm) and reactive to light. Neck holding time also improved to 12 sec; power improved to 5/5 in the left upper and lower limb and 3/5 in the right upper and lower limb. On day 30 of hospitalisation, the patient was doing well; he was discharged (GCS=E4V5M6) and referred for psychiatric consultation and physiotherapy.

## Discussion

Organophosphate exposure is a significant cause of pesticide-related mortality, with nearly 200,000 deaths annually [5]. Amongst the OPs, monocrotophos poisoning has the highest fatality rate (23.8%) and need for mechanical ventilation (87.3%). It is also categorised as a class 1 substance by WHO owing to its severe toxicity [6]. Monocrotophos is also known to cause intermediate syndrome, the prevalence of which is 20-40% of all OP poisoning [7,8]. We report a case of monocrotophos-induced intermediate syndrome complicated by hyperammonemic encephalopathy leading to difficulty in ventilator weaning. This report also highlights the need for early initiation of lactulose in monocrotophos-induced encephalopathy for more effective ventilator weaning and early extubation. 

Hyperammonemia is a common cause of encephalopathy characterized by episodic confusion and coma. It is usually seen in the setting of decompensated chronic liver failure, where the diagnosis is straightforward because of the other signs of chronic liver failure. However, there are other causes of hyperammonemia of non-hepatic origin presenting with neurological manifestations. These cases usually do not have any accompanying clinical signs, and the diagnosis often remains at bay, especially if the patient is sedated and under mechanical ventilatory support, wherein clinical signs of hepatic encephalopathy like asterixis cannot be elicited. 

**Figure 1:**
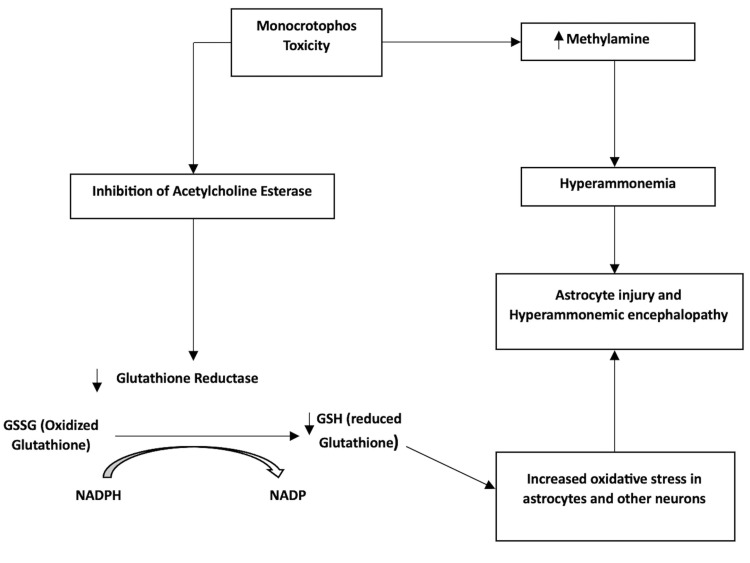
Monocrotophos metabolism, its interaction with glutathione (GSH) redox pathway, and neurotoxicity (hyperammonemic encephalopathy) NADPH: nicotinamide adenine dinucleotide phosphate hydrogen; NADP: nicotinamide adenine dinucleotide phosphate Figure by authors.

In this case, we hypothesize that one of the metabolites of monocrotophos is ammonia [9]. Though the liver functions were normal, the excess ammonia generated from the biodegradation of monocrotophos might have oversaturated the liver’s excretory capacity resulting in hyperammonemia precipitating encephalopathy. In addition, due to atropinization, the patient was constipated, which added more insult to injury by providing ample time for gut flora to degrade monocrotophos and generate ammonia. Another potentiating factor could be monocrotophos-induced acetylcholinesterase inhibition. This results in the suppression of glutathione reductase, an essential enzyme for reduced glutathione (GSH) synthesis [10,11]. GSH is pivotal in maintaining the antioxidant milieu inside the astrocytes, especially in the setting of hyperammonemia. It further enhances the deleterious effect of ammonia on neuronal cells, as in this patient. We have summarised the interaction of monocrotophos with glutathione redox pathway and hyperammonemia-induced neurotoxicity in Figure 1. 

We had started the patient on an osmotic laxative (lactulose) along with rifaximin, which relieved constipation. Later, L-ornithine L-aspartate was also added to the existing anti-encephalopathy measures, which led to a dramatic improvement in the patient's sensorium. Serum ammonia levels also showed a decreasing trend, which was clinically correlated with improving the patient's sensorium. Nonhepatic hyperammonemic encephalopathy is a diagnosis of exclusion; in this patient, we did not find any other cause of hyperammonemia (chemotherapeutic agents, infections like *Escherichia coli*, proteus, starvation, trauma, and antiepileptics) [12]. There was no past history or family history suggestive of urea cycle disorder. However, we could not rule out the possibility of subtle enzymatic defects, which might have contributed to hyperammonemia in addition to other factors. Moreover, the temporal correlation between serum ammonia levels and clinical improvement further substantiated our diagnosis. Timely identifying the underlying aetiology of non-hepatic hyperammonemia and expedited therapy may be lifesaving. Hence, a basic understanding of ammonia production and metabolism plays a significant role in evaluating and managing such patients.

## Conclusions

Neuropsychiatric complications in organophosphorus toxicity are not uncommon. However, metabolic encephalopathy is a rare occurrence. This case highlights hyperammonemic encephalopathy as an unusual, unexpected, fatal complication secondary to monocrotophos poisoning. It warrants a high index of clinical suspicion as it can be easily missed due to its similarity with other common neurological complications of organophosphate compounds. Serum ammonia levels should be monitored in cases with monocrotophos toxicity, especially in patients with altered sensorium.
